# Significance of myeloperoxidase plasma levels as a predictor for cardiac resynchronization therapy response

**DOI:** 10.1007/s00392-020-01690-1

**Published:** 2020-06-20

**Authors:** A. Sultan, J. Wörmann, J. Lüker, J. -H. v. d. Bruck, T. Plenge, V. Rudolph, A. Klinke, J. Heijman, M. Mollenhauer, T. Ravekes, S. Baldus, D. Steven

**Affiliations:** 1 Department of Electrophysiology, Heart Center, University of Cologne, Cologne, Germany; 2 Department of Cardiology, Heart and Diabetes Center North-Rhine Westphalia, Bad Oeynhausen, Germany; 3 Department of Cardiology, Maastricht University, Maastricht, The Netherlands; 4 Department of Cardiology and Cologne Cardiovascular Research Center (CCRC), Heart Center, University of Cologne, Cologne, Germany

**Keywords:** Cardiac resynchronization therapy, Response, Myeloperoxidase, Non-response, Inflammation

## Abstract

**Objectives:**

This study aimed to determine if changes in myeloperoxidase (MPO) levels correlate with response to cardiac resynchronization therapy (CRT) and the potential role of MPO as a predictor of response to CRT.

**Background:**

CRT is a well-established treatment option in chronic heart failure (CHF) with 50–80% of patients benefiting. Inflammation and oxidative stress play a key role in CHF pathophysiology. Previous studies have demonstrated increased levels of MPO in CHF patients, but the correlation with CRT response remains incompletely understood.

**Methods:**

Fifty-three patients underwent CRT implantation. During follow-up, patients were divided into two groups, responders and non-responders to CRT, based on improved physical capacity and NYHA classification. Levels of MPO and NT-pro-brain-natriuretic-peptide (NT-proBNP) were determined prior to implantation, 30 and 90 days after. Physical capacity, including a 6-min walking-test, NYHA class, and LVEF were evaluated at baseline and during follow-up.

**Results:**

Thirty-four patients (64%) responded to CRT, showing improved physical capacity and LVEF. All responders revealed a significant decrease of MPO levels (503.8 ng/ml vs. 188.4 ng/ml; *p *< 0.001). Non-responding patients did not show any significant changes in clinical parameters or MPO levels (119.6 ng/ml vs. 134.3 ng/ml; *p *= 0.672) during follow-up. At baseline, physical capacity and NYHA class, as well as MPO levels differed significantly between both groups (*p *< 0.001). A ROC analysis identified an MPO cut-off value for response to CRT of 242 ng/ml with a sensitivity of 93.5% and specificity of 71.4%. There was a strong correlation between MPO and improvement of LVEF (Spearman’s rho: − 0.453; *p *= 0.005) and physical capacity (Spearman’s rho: − 0.335; *p *= 0.042).

**Conclusions:**

Response to CRT and course of MPO levels correlate significantly. MPO levels differ between responders and non-responders prior to CRT, which may indicate an additional value of MPO as a predictor for CRT response. Further randomized studies are required to confirm our data in larger patient cohorts.

## Introduction

 Chronic heart failure (CHF) is the major cause of mortality and morbidity in western society [1]. In patients with CHF and left bundle branch block (LBBB) with a QRS width of > 130 ms, cardiac resynchronization therapy (CRT) is an established therapy option with proven beneficial outcome [1, 2]. Despite improvement of implantation efficiency and device technology, a non-responder rate between 20 and 50% is still evident [3, 4]. Therefore, prediction of possible CRT response would be of great value. Previous trials (PROSPECT (echocardiographic parameters) or TARGET (speckle-tracking for LV lead placement)) already investigated clinical parameters that potentially predict CRT response [5, 6] but so far are not established as tools to guide CRT implantation. In the MADIT-CRT cohort, brain-natriuretic peptide (NT-pro-BNP) was an independent predictor for CRT response 1 month after implantation suggesting a role for reversed remodeling in CRT response [7]. Further trials aiming to establish NT-pro-BNP as a predictive marker produced significant results, but had poor sensitivity (62%) for CRT response; therefore, NT-pro-BNP could not be used as an effective preimplantation marker [8]. In the quest for alternative markers, previous studies have revealed that the heme protein myeloperoxidase (MPO), released by activated polymorphonuclear neutrophils and to a smaller extent by monocytes, is significantly increased in CHF patients irrespective of the etiology [9]. Through oxidation of endothelial-derived nitric oxide, MPO is promoting left-ventricular and atrial remodeling. Released MPO leads to activation of macrophages and reactive oxygen species releasing PMN, perpetuating inflammatory processes, and left-ventricular remodeling [10]. Furthermore, MPO levels correlate with a decrease in left ventricular ejection fraction (LVEF) and the severity of CHF [9]. MPO is also an independent predictor of 1-year mortality in patients with CHF and a risk factor for acute coronary syndrome (ACS) [11]. However, improvement of physical capacity and LVEF remain the most valuable parameters for CRT response.

Therefore, CHF markers aside from NT-pro-BNP and clinical parameters may be beneficial to better predict CRT response in the setting of CHF. In a small clinical trial, it has been shown that MPO levels decrease after CRT implantation, while the predictive value of MPO before CRT was limited [12]. Thus, our investigation aimed to assess in a single-center patient cohort the role of MPO as a potential predictive discriminator for CRT response and to compare its correlation to response with established markers such as NT-pro-BNP.

## Methods

### Study population

The study population consisted of 53 consecutive patients undergoing CRT implantation at the University Heart Center Cologne (Germany) between September 2014 and March 2016 for CHF and LBBB > 130 ms (according to the guidelines at that time). This cohort’s size was chosen to perform an explorative trial to form a hypothesis regarding the interaction between MPO and the response to CRT. Patients were excluded from the analysis if the QRS to left-ventricular lead delay was < 80 ms to assure optimal LV lead positioning according to data of the SMART-AV trial [13, 14]. In 51 patients, a CRT-Defibrillator and, in 2 patients, a CRT-Pacemaker were implanted. All patients obtained optimal medical treatment prior to implantation. The study population was followed up for 30 and 90 days after implantation for clinical parameters and biomarkers such as MPO and NT-pro-BNP. During follow-up, there was no change in the medication for CHF.

Our study complied with the Declaration of Helsinki and its amendments [15]. The local ethics committee approved the study (Document No. 16-010) and all patients provided written informed consent. All procedures and measures were performed in accordance with the relevant guidelines and regulations.

### Evaluation of clinical parameters and CRT response

Aside from patients’ assessment, the response to CRT was defined as an increase in walking distance (in meters) in the 6-min walking test and improvement of ≥ 1 level in NYHA classification. Hospitalization or death due to CHF during follow-up was also defined as non-response to CRT. This definition of response to CRT focusing on symptomatic response is in line with the previous trials [7, 8]. LVEF was assessed using transthoracic echocardiography (Philips iE33 xMatrix Ultrasound Systems, Philips, The Netherlands), but did not influence classification as responder or non-responder due to our symptom-based definition of response.

All 6-min walking tests were performed indoor and under constant conditions. After evaluation of the covered walking distance, an assessment of patients’ capacity perception using a BORG’s scale was performed. Therefore, patients were asked to rate their degree of dyspnea during the exercise based on BORG’s scale ranging from 0 (no dyspnea) to 10 (maximal dyspnea).

All tests were repeated at 30 and 90 days after implantation at our facility, followed by reevaluation of the NYHA class level and LVEF.

### MPO and NT-pro-BNP measurements

As a main aspect in this study, we investigated a possible correlation between the courses of MPO levels and clinical response to CRT therapy. Therefore, baseline parameters of MPO and NT-pro-BNP levels, physical capacity, LVEF, and NYHA class were determined and analyzed to identify a possible correlation with CRT response and optimal MPO cut-off values for identifying responders. For MPO and NT-pro-BNP assessment, blood samples were taken prior to implantation, 30 and 90 days after implantation. Two milliliters (ml) of heparinized plasma was separated and stored at − 80 °C until completion of a full marker kit to undergo testing. For evaluation of MPO levels, the “Human Myeloperoxidase Quantikine ELISA-Kit No. DMYE00B” (R&D Systems Minneapolis, USA) was used. The quantity of NT-pro-BNP was measured using the “Cobas E602” pro-BNPII Immunoassay” (Roche Diagnostics, Switzerland).

### Statistical analysis

Statistical analysis was performed using IBM SPSS Statistics 23 (IBM, Armonk New York, USA). A *p* value < 0.05 was considered statistically significant.

Due to not normally distributed values of MPO and NT-pro-BNP, non-parametric tests (Mann–Whitney *U* test) were performed. Furthermore, we performed parametric tests, as our study design is hypotheses generating and not to determine a clinically used cut-off-value.

A post hoc analysis (LSD) was performed to compare changes in walking distance, LVEF, and NYHA class at baseline and during follow-up. Furthermore, quantile–quantile (Q–Q) plot, and Levene’s and Mauchly’s test were performed.

To assess the correlation between MPO and clinical parameters, Spearman’s rho and Pearson correlation coefficients were used. To reveal a potential prognostic value of MPO for CRT response, responder-operating curve (ROC) analysis was employed.

## Results

### Study population

A total of 53 patients (44 men, age 68 ± 13 years) with CHF and LBBB underwent successful CRT implantation. The majority of patients (*n *= 34 [64%]) suffered from ischemic heart disease and 19 (36%) from non-ischemic cardiomyopathy. All patients received their tolerated optimal medical treatment for CHF in accordance with current guidelines and fulfilled guideline criteria to undergo CRT implantation. There was no change in medication during follow-up. All patients had optimal biventricular stimulation (99.2% ± 1.0%). Within the study population, no differences regarding baseline parameters and medication were detectable (Table 1).

**Table 1:** Baseline characteristics of study population divided into responders (Rpts, *n *= 34) and non-responders (NRpts, *n *= 19) based on symptomatic improvement

Baseline characteristics of study population	*n *= 53	Rpts *n *= 34	NRpts *n *= 1	*p* value
Age (years)	68 ± 13	68 ± 13	68 ± 14	0.924
Ischemic heart disease (*n* [%])	34 [64%]	22 [65%]	12 [63%]	0.912
Non-ischemic heart disease (*n* [%])	19 [36%]	12 [35%]	7 [37%]	0.912
Atrial fibrillation (n [%])	22 [41.5%]	15 [44.1%]	7 [36.8%]	0.614
LV-EF (%)	27.0 ± 5.9	27.8 ± 6.3	26.1 ± 4.7	0.437
QRS (ms)	161.2 ± 21.0	163.7 ± 23.0	158.3 ± 19.2	0.422
NYHA III + IV (*n* [%])	42 [79%]	30 [88%]	12 [63%]	0.043
BMI (kg/m^2^)	28.2 ± 7.1	27.8 ± 7.1	29.0 ± 6.9	0.581
Hypertension (*n* [%])	36 [67.9%]	24 [70.6%]	12 [63.2%]	0.587
Diabetes (*n* [%])	22 [41.5%]	14 [41.2%]	8 [42.1%]	0.949
Beta blockers (*n* [%])	52 [98.1%]	34 [100%]	18 [94.7%]	0.184
ACE inhibitors (*n* [%])	43 [81.1%]	29 [85.3%]	14 [73.7%]	0.309
ARBs (*n* [%])	10 [18.9%]	5 [14.7%]	5 [26.3%]	0.309
MRAs (*n* [%])	49 [92.5%]	32 [94.1%]	17 [89.5%]	0.548
Diuretics (*n* [%])	52 [98.1%]	34 [100%]	18 [94.7%]	0.184
Amiodarone (*n* [%])	9 [17.0%]	3 [8.8%]	3 [15.8%]	0.866

*LVEF* left-ventricular ejection fraction, *QRS* duration of QRS in ms, *BMI* body mass index, *ARB* angiotensin II receptor blocker. Mean ± SD

### Response to CRT implantation and MPO levels

In 34 out of 53 patients (64%), there were a positive measurable response to CRT with an increase in walking distance and a decrease in NYHA class. Responders also had a significant increase in LVEF. Assessment of MPO levels and clinical parameters revealed that patients ultimately defined as responders based on clinical response had higher baseline levels of MPO than non-responders (503.8 ng/ml [315.9–768.7 ng/ml] vs. 134.3 ng/ml [78.8–300.1 ng/ml]; *p *< 0.001). In addition, at baseline, responders covered a shorter walking distance (145.7 ± 199.9 m vs. 293.4 ± 264.4 m; *p *= 0.021) and suffered more often from NYHA class III or IV (*n* = 30 [88%]) as compared to non-responders (12 [63%]; *p *= 0.043).

### Walking distance, NYHA class, and LVEF changes during follow-up

Patients in the responder group showed a significant increase in walking distance (145.7 ± 199.9 m vs. 308 ± 203 m; *p *= 0.004) within 30 days after CRT implantation, which was not observed in non-responders (293.4 ± 264.4 m vs. 289.7 ± 259.9 m; *p *= 0.959).

After 90 days, no further improvement in walking distance compared to 30 days of follow-up was seen in either responders (308.1 ± 203.4 m vs. 349.8 ± 175.1 m; *p *= 0.518) or non-responders (289.7 ± 259.9 m vs. 369.3 ± 248.4 m; *p *= 0.346).

A decrease in NYHA class was detected in 30 responders [88%] (*p *< 0.001) after 90 days.

Non-responders did not show any significant change in walking distance (293.4 ± 264.4 m vs. 369.3 ± 248.4 m; *p *= 0.369), or NYHA class (1 patient worsened; *p *= 1.000) during the entire follow-up, as expected by the definition of non-response. After 90 days, a significant increase in LVEF was detectable in the responders (27.8 ± 6.3% vs. 34.6 ± 10.6%; *p *= 0.001), but not in the non-responders (26.1% ± 4.7 vs. 27.8% ± 8.6; *p *= 0.554). Accordingly, after 90 days, responders showed a significantly higher LVEF than non-responders (34.1% ± 10.8 vs. 27.8 ± 8.6%; *p *= 0.023).

### Time course of MPO plasma levels

In the responders, MPO levels showed a significant decrease within 30 days after CRT implantation (503.8 ng/ml [315.9–768.7 ng/ml] at baseline vs. 215.5 ng/ml [84.3–483.1 ng/ml] at 30 days; *p *< 0.001). After 90 days, MPO levels declined further (188.4 ng/ml [108.6–395.3 ng/ml]; *p *< 0.001); however, this decrease did not reach statistical significance compared to the data obtained after 30 days.

**Fig. 1:**
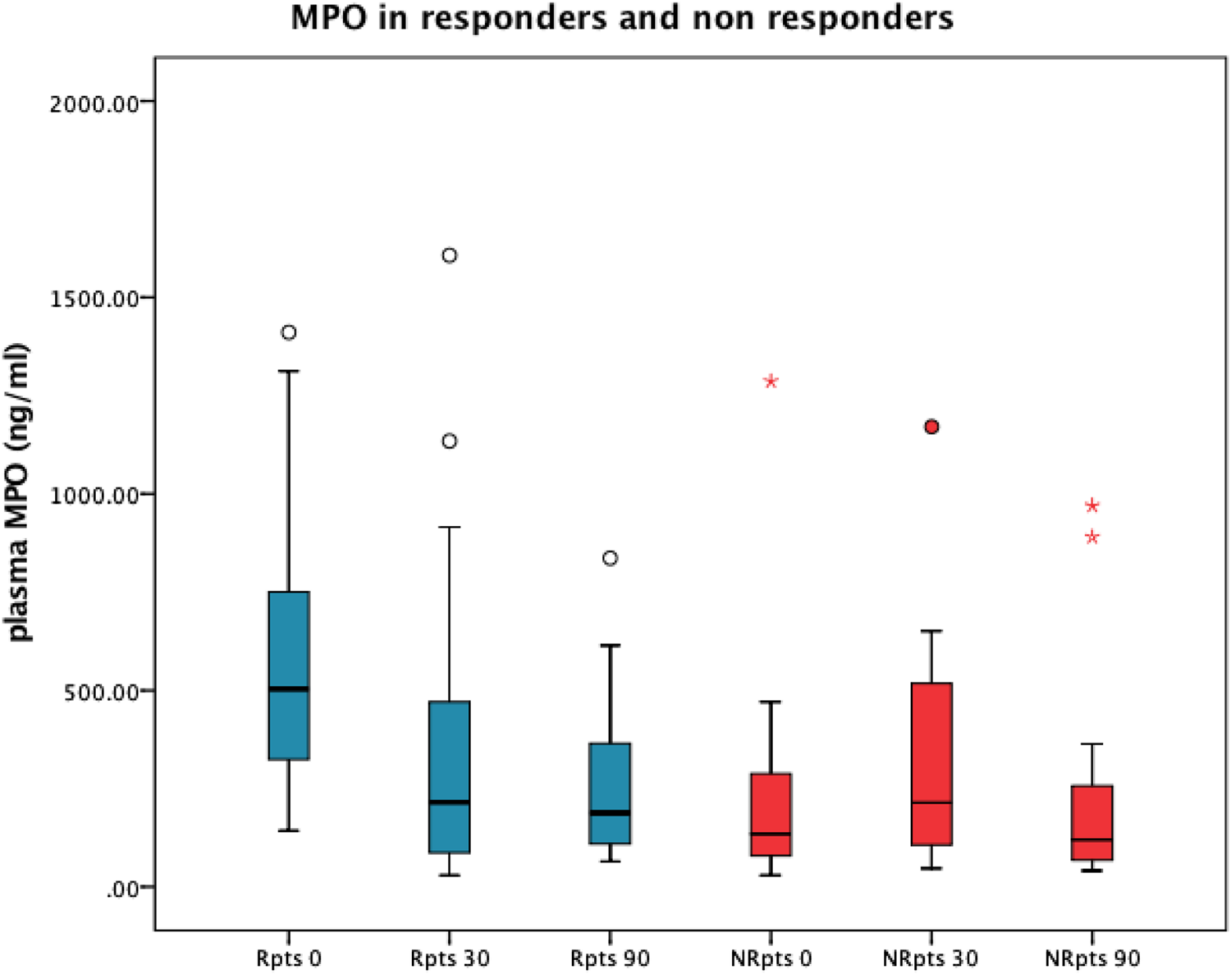
Boxplot of MPO plasma levels at baseline and during follow-up (0–90 days) after CRT implantation in responders (blue, *n *= 34) and non-responders (red, *n *= 19). Patients with elevated MPO levels at baseline revealed a significant decline in circulating MPO following CRT implantation and improvement in heart failure symptoms whereas those without elevated MPO did not profit from CRT implantation. Points indicating outliers. Asterisks indicate extreme outliers (three times higher than boxes)

Again, for non-responders, no significant change in MPO plasma levels was detectable after 90 days compared to baseline (134.3 ng/ml [78.8–300.1 ng/ml] vs. 119.6 ng/ml [66.3–363.7 ng/ml]; *p *= 0.672) (Fig. 1; Table 2).

**Table 2:** MPO and NT-pro-BNP levels and PC parameters over time and significance of means between response groups

	Rpts	NRpts	p R vs NR
0d			
MPO (ng/ml)	504 (316-769)	134 (79-300)	<0.001
WD (m)	146 ± 200	293 ± 264	0.021
EF (%)	28.4 ± 6.1	26.1 ± 4.7	0.437
NYHA III + IV (n[%])	30 [88%]	12 [63%]	0.043
NTpBNP (ng/ml)	2380 (854-6570)	2008 (1151-3854)	0.707
30d			
MPO (ng/ml)	216 (84-483)	215 (101-528)	0.626
WD (m)	308 ± 203	290 ± 260	0.776
NYHA III + IV (n[%])	7 [20%]	13 [68%]	0.001
90d			
MPO (ng/ml)	188 (109-395)	120 (66-364)	0.328
WD (m)	350 ± 175	369 ± 248	0.816
EF (%)	34.1 ± 10.8	27.8 ± 8.6	0.028
NYHA III + IV (n[%])	5 [15%]	13 [68%]	<0.001
NTpBNP (ng/ml)	2257 (574-4531)	1885 (1036-3696)	0.933

Data are shown as Mean ± SD or median (interquartile range) for not normally distributed values

*Rpts* responders, *NRpts* non-responders, *WD* walking distance in 6-minute walk test, *LVEF* left-ventricular ejection fraction, *0d* baseline, *30d* 30 days after implantation, *90d* 90 days after implantation

*p* < 0.05 was considered statistically significant

### Course of NT-pro-BNP and leukocyte count

At baseline, levels of NT-pro-BNP did not show any significant difference between groups (responders: 2380 ng/ml [854–6570 ng/ml]; non-responders: 2008 ng/ml [903–6473 ng/ml]; *p *= 0.774). Similarly, during follow-up, no significant changes in NT-proBNP levels compared to baseline values were detectable for any group (responders: 2257 ng/ml [574–10129 ng/ml] at 90 day follow-up; *p *= 0.364; non-responders: 1885 ng/ml [1036–3696 ng/ml] at 90 day follow-up, *p *= 0.708).

Leukocyte count was equal in both groups at baseline (responders: 7.7/nl ± 2.0/nl; non-responders: 7.6/nl ± 1.6/nl; *p *= 0.689). During follow-up, there was no significant change in leukocyte counts in both groups (responders: 7.5/nl ± 2/nl, *p *= 0.287; non-responders: 7.5/nl ± 1.6/nl, *p *= 0.689).

### Correlation between MPO, NT-pro-BNP, and response parameters

A significant correlation between MPO and NT-pro-BNP (Spearman’s rho: 0.198; *p *= 0.042) was detectable in both groups. Similarly, MPO plasma levels correlated with walking distance (Spearman’s rho: − 0.340; *p *= 0.04), and there was a strong correlation between relative change of MPO and improvement of LVEF (delta-MPO and delta-LVEF, Spearman’s rho: − 0.453; *p *= 0.005).

Furthermore, we identified a significant correlation between an improvement in NYHA class and a change in MPO levels (delta-NYHA and delta-MPO: Spearman’s rho: − 0.335; *p *= 0.042). These correlations could not be shown for NT-pro-BNP (delta-EF: Spearman’s rho: − 0.880; *p *= 0.333; delta-NYHA: Spearman’s rho: 0.161; *p *= 0.266).

### Diagnostic value of MPO in CRT patients

**Fig. 2:**
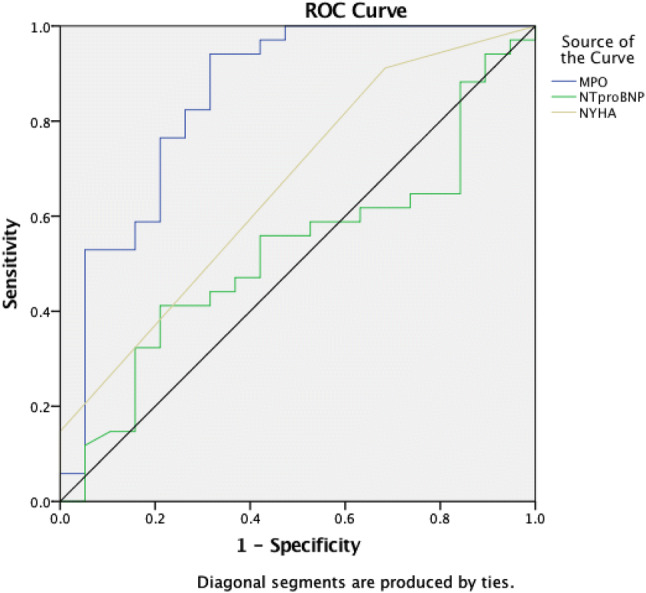
ROC curve displaying diagnostic accuracy for response and non-response to CRT for baseline plasma MPO level (blue), baseline NT-pro-BNP level (green), and baseline NYHA level (yellow). MPO showed an area under the curve of 0.85 (*p *< 0.001). An MPO level of 242 ng/ml or higher predicted a response with a sensitivity of 93.5% and a specificity of 71.4%

The ROC analysis revealed that MPO levels at baseline were the sole significant predictor for CRT response compared to baseline LVEF, NYHA class, or NT-pro-BNP levels, with an AUC of 0.85 (*p *< 0.001; Fig. 2).

In this small-study population, these data revealed that an MPO value of ≥ 242 ng/ml predicted CRT response with a sensitivity of 93.5% and a specificity of 71.4%

### QRS duration and biventricular stimulation

Baseline QRS duration did not differ significantly between the groups. Responders showed a mean QRS duration of 163.7 ms ± 23.0 ms, while non-responders had a slightly shorter QRS duration (158.3 ms ± 19.2 ms; *p *= 0.422) reflecting a guideline-based patient selection for CRT implantation. There was no difference in biventricular stimulation between both groups (responders: 99.2% ± 1.0%; non-responders: 99.1% ± 0.9%; *p *= 0.787).

## Discussion

### Main findings

This study aimed to evaluate the role of MPO in CRT recipients and its correlation to CRT response. As a main finding, this study revealed that MPO, which is known to be elevated in CHF, decreased significantly in patients who responded to CRT implantation based on objective clinical parameters (NYHA and walking distance) within 90 days after successful device implantation. For patients who did not show any clinical response to CRT, no significant changes in MPO levels were detectable, despite optimal CRT placement. These differences between responders and non-responders were independent of leukocyte count.

Baseline MPO levels differed significantly between those patients with CRT response as opposed to patients not responding to CRT. For this small-study cohort, an MPO level of 242 ng/ml or higher at baseline predicted a response after successful CRT implantation with a sensitivity of 93.5% and a specificity of 71.4%.

A significant correlation between reverse remodeling (improvement of LVEF, *p *= 0.005) and physical capacity (NYHA improvement, *p *= 0.042) was shown for MPO, while NT-pro-BNP did not correlate significantly with response to CRT in our cohort.

### Significance of MPO levels

Different levels in baseline MPO values in patients with CHF and eligible for CRT presumably reflect distinct types or severity of CHF and structural remodeling in these patients. Beside LVEF, impaired physical activity and NYHA class often only partially describe the severity of CHF without sufficient discrimination. Baseline levels of MPO might be a more objective parameter to sub-stratify the patient population with CHF. Therefore, markedly elevated baseline MPO levels possibly reflect a more advanced stage of CHF.

In contrast, in patients with low MPO levels at baseline, the severity of CHF and structural remodeling may be less pronounced and, therefore, CRT implantation in these patients might be less beneficial although fulfilling CRT implantation criteria [9, 10].

### The role of MPO as a predictor

To distinguish responders from non-responders before CRT implantation, it would be desirable to have additional tools complementing established clinical parameters such as LVEF and LBBB width [1]. As mentioned before, evaluation of MPO levels seems to be of value for a thorough assessment of CHF severity and potentially predicting CRT response in these patients. This study revealed a significant correlation between MPO levels and CRT response.

However, to use MPO as a predictor for CRT response before implantation in patients with CHF, a cut-off value would have to be identified. Therefore, in this explorative trial with a small single-center cohort, ROC analysis suggested that MPO values of 242 ng/ml or higher predicted a CRT response with a high sensitivity of 93.5% and an acceptable specificity of 71.4%. This analysis showed that MPO was the only significant parameter for CRT response in comparison with NT-pro-BNP and NYHA class. These findings support the hypothesis that MPO could be used as a preimplantation marker to reveal possible non-responders.

Regarding the role of MPO in the etiology of CHF, promoting increased structural remodeling, high MPO levels may predict a better response to CRT indicating advanced oxidative stress [9, 10, 16]. The role of CRT for a reversed structural remodeling has been proven in the REVERSE trial [17]. The fact that, in our analysis, MPO correlated with NT-proBNP, a strong parameter for structural remodeling in heart failure, implies that MPO levels decrease because of reverse remodeling under successful CRT.

Considering improvement of myocardial performance due to CRT (increase of LVEF) strongly correlates with decreasing MPO levels in CHF patients, our data imply that this improvement is promoted by anti-inflammatory effects as described before in several studies [10, 18, 19].

The effect of hemodynamic improvement in CHF patients on MPO levels has been shown in the previous trials with levosimendan, a drug used in the setting of acute heart failure or worsening of CHF. Blood pressure regulation directly affects PMN activation and endothelial MPO deposition [20]. The positive effects of CRT on hemodynamics in CHF patients possibly lead to a better MPO clearance. Latter might explain the revealed effects in our cohort.

Furthermore, our data show that MPO may be more sensitive as a clinical marker for CRT response than NT-proBNP. Though, it should be acknowledged that multivariate analysis in larger cohorts will have to prove the incremental benefit compared to clinical markers as LVEF and NYHA.

Previous studies with larger populations have shown that additional assessment of MPO to NT-proBNP was able to increase specificity in heart failure patients (74.1% vs. 40.5% with NT-proBNP alone) [21]. In contrast to earlier studies, we were not able to confirm a decrease of NT-pro-BNP in CRT responders [8, 22]. Also, in our cohort there was no correlation between CRT response and the course of NT-pro-BNP. This might be explicable by differences between study populations and follow-up duration. Thus, the patient cohort in the study of Lellouche et al. did not include patients with NYHA class < 3 and follow-up was longer than in our cohort [8]. We assume that MPO is the more sensitive and, therefore, prompt marker because of its direct mechanistical link to improve hemodynamic in CRT patients. Furthermore, in these trials, NT-proBNP has not been proven to be a sufficient preimplant marker for CRT, due to poor sensitivity of 62% [7, 8].

A previous trial with 44 pts by Sunman et al. also investigating the course of MPO showed that MPO levels decrease after CRT implantation consistent with our data. In this analysis, the investigators could not find significant differences between responders and non-responders at baseline and during follow-up. However, the goal of that study was to correlate MPO and other cardiac biomarkers. There was no investigation of the correlation between clinical parameters and MPO [12].

The major aspect why MPO suggests being superior to other markers of oxidative stress is that it is mechanistically linked to the development of heart failure. A major driver in this regard seems to be oxidation of endothelial-derived nitric oxide.

In contrast, other markers like MDA and isoprostanes simply reflect the burden of oxidative stress which might have a higher variability, lower organ specificity, and not a direct link to vascular and or myocardial function. Therefore, we believe that MPO with its profound body of evidence for impacting on vascular function is not only marker but potentially mediator of the disease, which makes it such a powerful indicator of changes in LV function [10]..

### Limitations

There are a few limitations to this study that should be mentioned. Our cohort was relatively small. Larger studies are required to identify a valid cut-off value, while our investigation sought to evaluate the hypothesis of MPO as a possible predictor. Furthermore, a multivariate analysis could be performed in a larger cohort to assess the predictive. Presented data and evaluated MPO levels including a cut-off value reflect a single-center experience. For a cut-off value used in wide clinical practice, prospective trials should be performed. However, the patients’ cohort presented here represents a typical population of CRT recipients undergoing guideline-based therapy.

## Conclusion

This study demonstrates that MPO levels decrease in CRT recipients responding to the therapy. Furthermore, change in MPO levels correlates significantly with the development of parameters of cardiac remodeling and clinical capacity, which we could not show for NT-pro-BNP. In our cohort, patients with MPO levels higher than 242 ng/ml at baseline did respond to CRT within 90 days after implantation, indicating that a possible cut-off value could be found in a larger population. Further prospective evaluation of MPO as a therapy denominator is required and studies are planned and underway to address this aspect.
